# Fixed drug eruption after the Sinopharm COVID‐19 vaccine

**DOI:** 10.1002/jvc2.40

**Published:** 2022-06-29

**Authors:** Mahsa Rekabi, Elham Sadati, Jamal Mirzaei, Guitti Pourdowlat, Ali Akbar Velayati, Parisa Honarpisheh

**Affiliations:** ^1^ Allergy and Immunology Department, Pediatric Respiratory Diseases Research Center, National Research Institute of Tuberculosis and Lung Diseases (NRITLD) Shahid Beheshti University of Medical Sciences Tehran Iran; ^2^ Infectious Disease Research Center Shahid Beheshti University of Medical Sciences Tehran Iran; ^3^ Chronic Respiratory Diseases Research Center National Research Institute of Tuberculosis and Lung Diseases (NRITLD), Shahid Beheshti University of Medical Sciences Tehran Iran

**Keywords:** BBIBP‐Cor, COVID‐19 vaccine, fixed drug eruption, side effects, Sinopharm

## Abstract

After coronavirus disease 2019 (COVID‐19) became widespread around the world, several vaccines have been developed with variable efficacy and potency and based on different platforms to control the pandemic. One of these vaccines is Sinopharm (BBIBP‐CorV), which is an inactivated virus that was released by Sinopharm's Beijing institute in the summer of 2020. The most commonly reported side effects of the Sinopharm vaccine have included pain at the injection site, muscle pain, headache and fatigue. Dermatological reactions are also reported as less common and were mainly local injection site reactions. Fixed drug eruption (FDE) is a rare and unusual adverse effect and accounts for less than 1% of all severe acute respiratory syndrome coronavirus 2 vaccine‐related cutaneous manifestations. FDE has not been reported following the COVID‐19 inactivated vaccine. Here, we describe a rare case of FDE following the administration of the first shot of the Sinopharm vaccine.

## INTRODUCTION

As with any other vaccines, side effects are expected after coronavirus disease 2019 (COVID‐19) vaccines; so far, most of these have been mild and self‐limited. A wide spectrum of skin manifestations has occurred among coronavirus vaccine recipients, which appear to be more common with the Moderna vaccine.^1^ Injection site reactions are the most common cutaneous reaction to COVID‐19 vaccines. Fixed drug reaction (FDE) is a rare skin side effect previously reported in eight cases after COVID‐19 vaccines, but none of them after the Sinopharm vaccine. We present here a 38‐year‐old Iranian woman who developed FDE after the Sinopharm vaccine.

## CASE PRESENTATION

**Figure 1:**
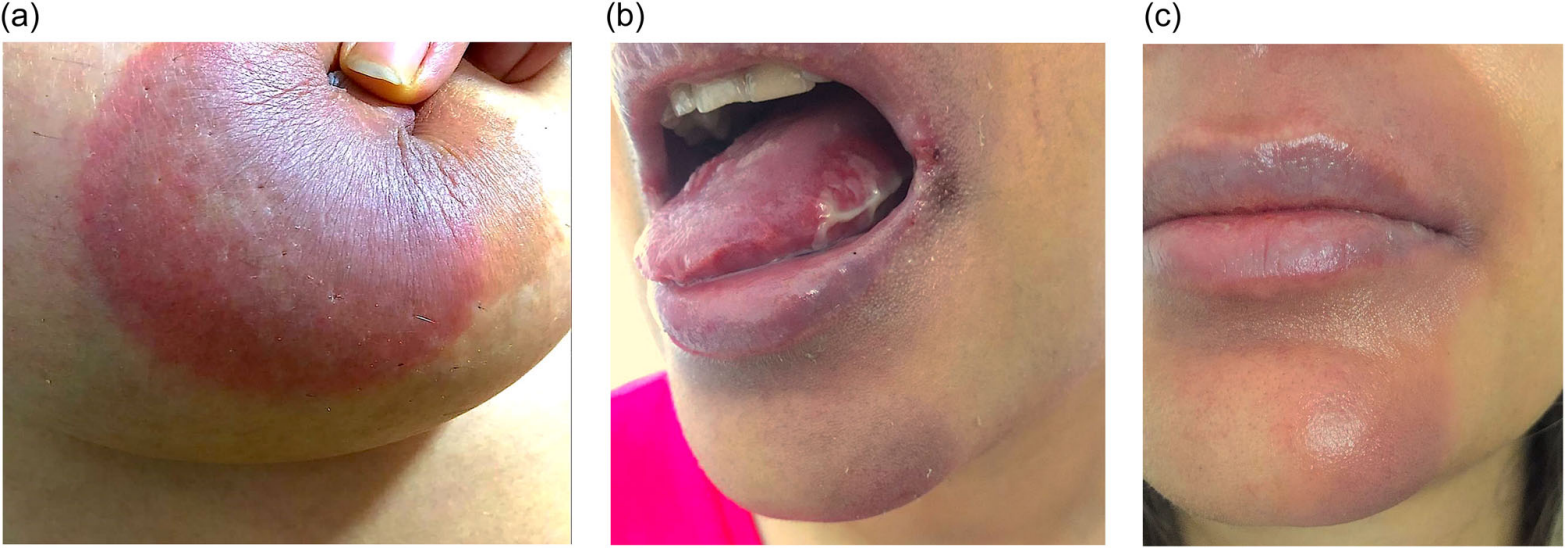
Fixed drug eruption (FDE) following Sinopharm vaccine. (a) An erythematous plaque on the breast. (b) Mucosal involvement by FDE. (c) Hyperpigmentation of perioral region.

A 38‐year‐old woman was admitted to the allergy and immunology clinic of our hospital because of skin lesions that had begun 24 h after receiving the first dose of the Sinopharm vaccine. The lesions were slightly itchy and painful. Physical examination demonstrated well‐defined erythematous patches located on the legs, hands, feet, breasts, genital area, anus and around the mouth. There was also inflammation and ulceration of the buccal mucosa. The lesions varied in size and did not disappear with pressure. Ten days later, her skin lesions improved leaving residual hyperpigmentation (Figure 1). After receiving the second dose of the Sinopharm vaccine, the lesions reappeared within half an hour and with the same characteristics on the back, feet, hands and around the mouth. No constitutional or systemic manifestations were observed. The patient had not taken any medication and had no history of food allergy, prior drug or vaccine‐induced reactions or underlying disease. The laboratory investigations included complete blood count, erythrocyte sedimentation rate, C‐reactive protein and rheumatologic tests (fluorescent antinuclear antibody, anti‐double‐stranded DNA antibody, anti‐cyclic citrulline peptides antibody, perinuclear antineutrophil cytoplasmic antibodies and antineutrophil cytoplasmic antibody) were within the normal range. A nasopharyngeal swab for coronavirus was also negative.

**Figure 2:**
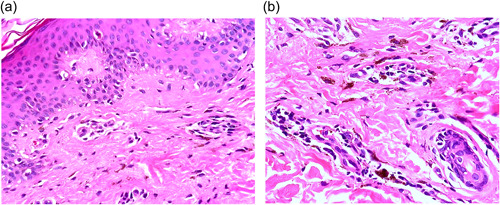
Histopathology of skin biopsy. (a) The epidermis showed hyperkeratosis, dyskeratotic cells and vacuolar changes in the basal layer. The dermis revealed melanin incontinence, endothelial swelling and perivascular lymphocytic infiltration (H&E stain, original magnification: ×100). (b) Prominent melanin deposition in the dermis (H&E stain, original magnification: ×200). H&E, hematoxylin and eosin.

A biopsy was taken from the lesion that reappeared on the back, showing hyperkeratosis, dyskeratotic cells and vacuolar changes of the basal layer of the epidermis and perivascular infiltration of inflammatory cells, mainly lymphocytes, in the upper dermis. The reported pathological picture revealed that changes were consistent with the diagnosis of FDE (Figure 2). A topical corticosteroid was prescribed, and after 3 weeks, she reported complete resolution of her lesions with residual pigmentation in some involved areas.

## DISCUSSION

COVID‐19 vaccines' effectiveness, safety and side effects have been a matter of concern. A systematic review about the safety of different COVID‐19 vaccines showed that RNA‐based vaccines had the highest incidence of side effects, whereas inactivated vaccines had the lowest.^2^ In another systematic review of 229 articles,^1^ a total of 5941 cases with skin manifestations appeared following severe acute respiratory syndrome coronavirus 2 (SARS‐CoV‐2) vaccine administration; of these, local injection site reactions (34.05%) were the most common manifestations, followed by unspecified skin eruptions (32.88%), urticaria (10.89%) and angioedema (5.35%). Adverse effects have mainly occurred after the first dose of the vaccine.^3^ Dermatological reactions were found to be more common after the Pfizer and Moderna vaccines.^1^ Sinopharm, an inactivated virus SARS‐CoV‐2 vaccine, was developed in China. This vaccine induced mild, self‐limiting adverse reactions, most commonly pain at the injection site and fever. Allergy was seen in 1.1% of cases after the second vaccine shot versus 0.0% following the first dose of Sinopharm COVID‐19 vaccination.^4^ FDE was observed in eight (0.13%) cases; two of them appeared following the Pfizer vaccine, three after the Moderna vaccine and in three subjects the vaccine type was not specified.^1^ An additional case of FDE associated with the COVID‐19 vaccine has been reported in a 74‐year‐old man after the first dose of the Oxford–AstraZeneca vaccination.

FDE is considered a delayed hypersensitivity reaction. Although the exact mechanisms underlying allergic reactions after the vaccine are still unknown, the proposed process is an antibody and cell‐mediated response leading to the production of cytokines such as interferon‐gamma (IFN‐γ) and tumour necrosis factor‐alpha (TNF‐α), with an inflammatory effect on vessels, skin and other tissues.^5^

FDE is characterized by distinctive, well‐defined, erythematous or purplish lesions of the skin and mucosa. Several main variants of FDE exist, including pigmenting, nonpigmenting, bullous and mucosal types.^6^ FDE can be triggered by some foods (seafood, nuts, strawberry, kiwi and others) and drugs (nonsteroidal anti‐inflammatory drugs, antiepileptics such as phenytoin, antibiotics such as cotrimoxazole and others) and can be seen in all ages and both sexes. In the pigmentary variant, FDE gradually fades away leaving residual postinflammatory hyperpigmentation. Typically, FDE lesions reappear at the same sites after re‐exposure to the causative drug.^7^ The disease is usually mild and self‐limited and the primary treatment is identifying and cessation of the causative stimuli and conservative care.^8^

FDE can occur after using the Sinopharm vaccine. Because many side effects in the real‐world community setting may not be seen in clinical trials, reporting these complications is important for the clinical dermatologist.

## CONFLICT OF INTEREST

The authors declare no conflict of interest.

## ETHICS STATEMENT

The patient in this manuscript has given written informed consent to the publication of her case details. Ethical standards of the Shahid Beheshti University of Medical Sciences research committee (IR.SBMU.NRITLD.REC.1401.027).

## Data Availability

The data sets are available from the corresponding author on reasonable request.
